# Influence of Carbonization Conditions on Structural and Surface Properties of K-Doped Mo_2_C Catalysts for the Synthesis of Methyl Mercaptan from CO/H_2_/H_2_S

**DOI:** 10.3390/nano13182602

**Published:** 2023-09-21

**Authors:** Xiangqian Zheng, Tianhao Ai, Yuhong Hu, Zhizhi Xu, Yubei Li, Huan Jiang, Yongming Luo

**Affiliations:** 1 Faculty of Environmental Science and Engineering, Kunming University of Science and Technology, Kunming 650500, China; 2 Xishuangbanna Prefecture Comprehensive Inspection Center of Quality and Technical Supervision, Jinghong 666100, China; 3 Faculty of Chemical Engineering, Kunming University of Science and Technology, Kunming 650500, China; 4 Yunnan Research Academy of Eco-Environmental Sciences, Kunming 650093, China

**Keywords:** methyl mercaptan, CO/H_2_S/H_2_, molybdenum carbide, effect of carbonization conditions

## Abstract

The cooperative transition of sulfur-containing pollutants of H_2_S/CO/H_2_ to the high-value chemical methyl mercaptan (CH_3_SH) is catalyzed by Mo-based catalysts and has good application prospects. Herein, a series of Al_2_O_3_-supported molybdenum carbide catalysts with K doping (denoted herein as K-Mo_2_C/Al_2_O_3_) are fabricated by the impregnation method, with the carbonization process occurring under different atmospheres and different temperatures between 400 and 600 °C. The CH_4_-K-Mo_2_C/Al_2_O_3_ catalyst carbonized by CH_4_/H_2_ at 500 °C displays unprecedented performance in the synthesis of CH_3_SH from CO/H_2_S/H_2_, with 66.1% selectivity and a 0.2990 g·g_cat_^−1^·h^−1^ formation rate of CH_3_SH at 325 °C. H_2_ temperature-programmed reduction, temperature-programmed desorption, X-ray diffraction and Raman and BET analyses reveal that the CH_4_-K-Mo_2_C/Al_2_O_3_ catalyst contains more Mo coordinatively unsaturated surface sites that are responsible for promoting the adsorption of reactants and the desorption of intermediate products, thereby improving the selectivity towards and production of CH_3_SH. This study systematically investigates the effects of catalyst carbonization and passivation conditions on catalyst activity, conclusively demonstrating that Mo_2_C-based catalyst systems can be highly selective for producing CH_3_SH from CO/H_2_S/H_2_.

## 1. Introduction

Coal is the main energy source in China. This produces large quantities of sulfur-containing pollutants emitted by coal combustion, which has resulted in serious atmospheric environmental pollution [1,2,3]. Therefore, the removal and utilization of hydrogen sulfide resources have become research hotspots in the coal chemical industry [4,5]. In this industry, the Claus method and the improved Claus method are generally used to recover H_2_S to produce sulfur or sulfuric acid, and the added value of the products is low. The preparation of the high-value chemical methyl mercaptan (CH_3_SH) from H_2_S, along with CO and H_2_ in the process of coal gasification, has become a valuable development direction [6,7,8,9]. This technology can not only effectively remove H_2_S but also provide a new route for downstream product development. The key to realizing the efficient utilization of raw materials and the high yield of products is to build a high-performance catalyst system. Until now, molybdenum sulfide based catalysts have been widely preferred because of their excellent sulfur resistance, but the reactant conversion and product selectivity are poor [10,11], which greatly restricts the development and application of molybdenum sulfide based catalysts.

In the industry, CH_3_SH is mainly synthesized from the thiolation of methanol over alkali-promoted transition metal catalysts [12,13], but methanol needs to be prepared with syngas (CO + H_2_), which leads to complex processes, massive energy consumption and high economic costs. Olin et al. [14] developed a one-step synthesis method of CH_3_SH using syngas raw materials and reduced sulfur species (hydrogen sulfide) generated during coal gasification that will greatly reduce the production cost of CH_3_SH. Therefore, the preparation of CH_3_SH from CO/H_2_/H_2_S has received widespread attention in recent years [15,16,17], and most research has focused on tungsten-based and molybdenum-based catalysts.

Among these, sulfurized K-Mo-based materials have received widespread attention due to their good catalytic activity and excellent sulfur resistance [18,19,20,21]. Many studies have been focused on the reactive phase, the reaction path and improving the performance of the catalysts by regulating catalyst carriers, additives, preparation methods and so on. Liu et al. [7] found that the addition of alkali metals (Na, K, Cs) can lead to different sulfidation degrees of Mo oxidized species owing to the different electric effects of alkali metals, which result in the formation of different concentrations of alkali-metal-promoted Mo containing coordinatively unsaturated sites, dramatically improving the CH_3_SH selectivity and CO conversion. Lu et al. [22] synthesized mesoporous silica (SBA-15)-supported, 2–4 layered, ordered and K-promoted MoS_2_ nanosheets. K promotion played an important role by stabilizing the C-S bond during the adsorption of the intermediate, COS, to avoid its conversion to side products in the gas phase, so the CO conversion and the selectivity towards CH_3_SH were obviously improved. Yu et al. [23] also found not only that K can enhance the MoS_2_-catalyzed synthesis of methanethiol, whereby synergy increases with alkali cation size, but also that alkali sulfides themselves were the active sites. However, there are still obstacles in CO/H_2_/H_2_S synthesis of CH_3_SH using a molybdenum sulfide based catalyst, such as low reactant conversion and low selectivity towards the product, CH_3_SH. Therefore, it is still of great significance to explore high-performance catalysts.

The transition metal carbide (TMC) molybdenum carbide (Mo_2_C), which acts as an electron donor, exhibits high catalytic activity and selectivity due to the redistribution of electrons after carbon introduction. It has been widely applied for selective C-C, C-O and C-H bond cleavage and for producing fuel molecules from oxygenated biomass [24,25,26,27]. Moreover, it has good sulfur resistance and is expected to replace noble metal catalysts, which are limited by their low abundance and high cost [28,29]. The current synthesis methods of Mo_2_C including solid–gas reactions [30,31], solid–liquid reactions [32,33,34] and solid–solid reactions [35,36,37], among which solid–gas reactions are the most commonly used and involve the carbonization reaction of molybdenum oxide (MoO_3_ or MoO_2_) with hydrogen and alkane/aromatics. During the carbonization process, different carbonization atmospheres can lead to the formation of different crystal forms of Mo_2_C [27]. At present, there are no reports of the synthesis of CH_3_SH from CO/H_2_/H_2_S over molybdenum carbide based catalysts. Due to their unique structures, molybdenum carbide based materials are expected to become emerging catalysts for the synthesis of CH_3_SH from CO/H_2_/H_2_S.

Here, K-Mo/Al_2_O_3_ catalysts are prepared by the impregnation method, and a series of characterizations, including H_2_ temperature-programmed reduction (H_2_-TPR), Brunauer–Emmett–Teller (BET) analysis, temperature-programmed desorption (TPD), powder X-ray diffraction (XRD) and Raman spectroscopy, are conducted to investigate the effects of catalyst carbonization and passivation conditions on catalyst activity. We show that the regulation of carbonization and passivation conditions dramatically impacts the active sites and surface chemistry of the catalysts, and we explain how this can be exploited to greatly propel the production of CH_3_SH from CO/H_2_/H_2_S.

## 2. Experimental Section

### 2.1. Catalyst Preparation

K-Mo/Al_2_O_3_ catalysts with a Mo loading of 10 wt% were prepared by the impregnation method, wherein γ-Al_2_O_3_ was used as the support and ammonium molybdate tetrahydrate ((NH_4_)_6_Mo_7_O_24_·4H_2_O) and potassium carbonate (K_2_CO_3_) were used as the precursors of Mo and K, respectively. Firstly, a certain amount of (NH_4_)_6_Mo_7_O_24_·4H_2_O was fully dissolved in 3.5 mL deionized water, and then K_2_CO_3_ was added to the solution at a K:Mo molar ratio of 2:1. Next, 2 g support was added to the mixed solution and stirred. The mixture was allowed to stand overnight for aging and was then placed in an oven and dried at 110 °C for 6 h. Finally, the samples were transferred to a muffle furnace and heated from room temperature to 550 °C at 5 °C/min, with calcination in air for 5 h. The resulting catalyst was labelled as K-Mo/Al_2_O_3_.

### 2.2. Catalyst Characterization

Powder X-ray diffraction (XRD) of catalysts was performed on a Bruker Germany D8 ADVANCE instrument with Cu Kα-radiation (λ = 0.15418 nm) operating at 40 kV and 30 Ma. Raman spectroscopy (Jobin-Yvon France LabRam HR 800) was performed under 532 nm laser excitation, and the collection range was 200–1200 cm^−1^. BET surface areas and pore structures of catalysts were determined by N_2_ physisorption at 77 K using an America Quantachrome NOVA4200e analyzer. The temperature-programmed reduction of the sulfided sample (0.05 g) by hydrogen (H_2_-TPR) was performed using a thermal conductivity detector (TCD) at a heating rate of 10 °C/min until reaching 800 °C. The reduction atmosphere comprised a 10% H_2_/Ar (*v*/*v*) gas at a flow rate of 30 mL/min. The temperature-programmed desorption of carbon monoxide/hydrogen/hydrogen sulfide (CO/H_2_/H_2_S-TPD) was conducted in a fixed-bed flow reactor equipped with a thermal conductivity detector (TCD) as the detector. Taking CO-TPD as an example, prior to the analysis, the sample (0.05 g, 40–60 mesh) was first heated to 300 °C at a rate of 5 °C/min and kept at 300 °C for 0.5 h to remove adsorbed matter from the catalyst surface. After the sample was cooled down to room temperature, the gas stream was switched to 10% CO/He (30 mL/min). The adsorption was carried out at room temperature for 1 h, and then the sample was purged with Ar to remove the physically adsorbed CO. The reactor was heated from room temperature to 900 °C at a rate of 10 °C/min for temperature-programmed desorption. With other conditions remaining unchanged, H_2_-TPD and H_2_S-TPD were performed by replacing 10% CO/He with 10% H_2_/Ar and 10% H_2_S/He, respectively.

### 2.3. Catalyst Activity Test

#### 2.3.1. Catalytic Performance Tests

Before the performance test, 0.8 g K-Mo/Al_2_O_3_ (40–60 mesh) was placed in a quartz tube and a pre-treatment gas (carbonization gas: CH_4_/H_2_, C_2_H_6_/H_2_, C_3_H_8_/H_2_) was introduced. Taking CH_4_/H_2_ as an example, the ratio of CH_4_:H_2_ was 1:9 (*v*/*v*, 40 mL/min); the sample was first heated from room temperature to 300 °C under a carbonization atmosphere at a rate of 1 °C/min and was maintained for 2 h; the sample was then further heated to the carbonization temperature stage (400 °C, 500 °C, 600 °C) at a rate of 1 °C/min and held for 2 h. K-Mo/Al_2_O_3_ samples were carbonized in CH_4_/H_2_, C_2_H_6_/H_2_ or C_3_H_8_/H_2_ atmospheres and named as CH_4_-K-Mo_2_C/Al_2_O_3_ or K-Mo_2_C/Al_2_O_3_, C_2_H_6_-K-Mo_2_C/Al_2_O_3_ and C_3_H_8_-K-Mo_2_C/Al_2_O_3_, respectively.

The activity evaluation of the catalysts (0.4 g) was carried out on a fixed-bed reactor. A gas mixture of CO/H_2_/H_2_S (gas volume concentration: CO = 10%, H_2_ = 40%, H_2_S = 50%) was used. N_2_ was used as the equilibrium component and was introduced to the quartz tube with a pressure of 0.2 MPa and a flow rate of 40 mL/min. The catalytic activity was measured in the range of 275–400 °C. After reaching a steady state at each temperature, the products were analyzed online using three gas chromatographs (GC9790, China FULI INSTRUMENTS) equipped with FPD, TCD and FID.

#### 2.3.2. Analysis of the Products

The CO conversion rate and product selectivity were calculated using the following equation:

$${Con}_{CO}=\frac{C_{\mathrm{CO},\mathrm{in}}\;-\;C_{\mathrm{CO},\mathrm{out}}}{C_{\mathrm{in}}}\times 100\%\;{\mathrm{Sel}}_{X}=\;X_{i}/\sum_{i=1}^{n}X_{i}\times 100\%=\frac{C_{i}}{C\left({\mathrm{CH}}_{3}\mathrm{SH}\right)+C\left(\mathrm{COS}\right)+C\left({\mathrm{CO}}_{2}\right)+C\left({\mathrm{CH}}_{4}\right)+C\left({\mathrm{CS}}_{2}\right)}\times 100\%$$

where $C_{CO,in}$ denotes the concentration of CO in the feed gas and $C_{\mathrm{CO},\mathrm{out}}$ denotes the concentration of CO in the product; the product selectivity is calculated based on carbon balance, where i is the target product (CH_3_SH, COS, CO_2_, CH_4_, CS_2_). C_i_ is the product concentration.

The CO consumption rate and CH_3_SH formation rate were calculated using the following equation:

$$r\left(CO\right)=\frac{M_{CO}\times C_{CO,in}\times conCO\times Q}{V_{m}\times m_{cat}}\;r\left({CH}_{3}SH\right)=\frac{M_{{CH}_{3}SH}\times C_{{CH}_{3}SH}\times Q}{V_{m}\times m_{cat}}$$

where M_CO_ is CO molar mass (g/mol); M_CH3SH_ is the molar mass of CH_3_SH (g/mol). Q is the flow rate (L/h), V_m_ is the molar volume of gas under standard condition (L/mol) and m_cat_ is the catalyst amount (g).

## 3. Results

### 3.1. Performance of Catalysts Prepared at Different Carbonization Temperatures

**Figure 1:**
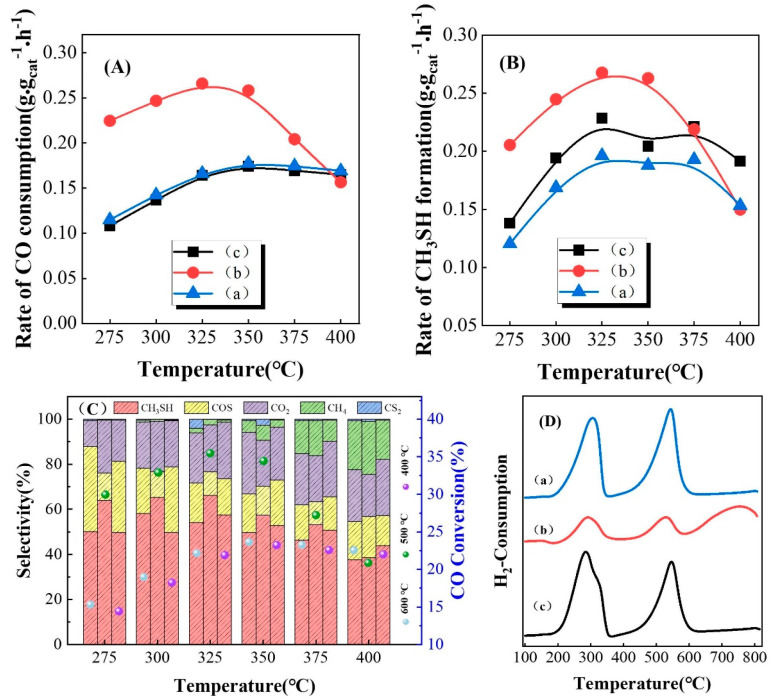
K-Mo/Al_2_O_3_ catalysts prepared with different carbonization temperatures were used to synthesize CH_3_SH from CO/H_2_/H_2_S; (a) K-Mo/Al_2_O_3_-600 °C; (b) K-Mo/Al_2_O_3_-500 °C; (c) K-Mo/Al_2_O_3_-400 °C. Rate of CO consumption (**A**); Rate of CH_3_SH formation (**B**); Distribution of CO conversion and main product selectivity (**C**); Curves of H_2_-TPR (**D**).

Figure 1 shows the effect of different carbonization temperatures on the catalytic performance of the K-Mo/Al_2_O_3_ catalyst for the synthesis of CH_3_SH from a CO/H_2_/H_2_S mixture. From Figure 1A,B, it can be seen that the CO consumption rate first increased and then decreased with increasing temperature over 325 °C. Our former studies showed that the equilibrium constant of the CO consumption reaction (CO + H_2_S → COS + H_2_) decreases with increasing temperature, which indicates that rising temperature is adverse to the occurrence of the CO consumption reaction. Therefore, the decrease in CO conversion at high temperatures is due to equilibrium limitations [7]. The CH_3_SH formation rate also showed a similar tendency to that of CO consumption. It can be obviously seen from the tendency that when the oxidation state material was carbonized at 500 °C, the catalyst was found to have the optimum catalytic activity, and the CO conversion (35.5%) and selectivity towards CH_3_SH (66.1%) reached maximum values when the reaction temperature was 325 °C. At this temperature, the CO consumption rate reached 0.2660 g·g_cat_^−1^·h^−1^ and the CH_3_SH formation rate reached 0.2990 g·g_cat_^−1^·h^−1^. Recently, Lu et al. investigated the synthesis of CH_3_SH using CO/H_2_/H_2_S as reactants over K-Mo-type catalysts. COS was demonstrated to be generated first via the reaction between CO and H_2_S (as shown by Equation (1)), and then CH_3_SH was formed via two reaction pathways, which were the hydrogenation of COS and CS_2_ (as shown in Equations (3) and (4)).

CO + H_2_S → COS + H_2_ (1)

COS + 3H_2_ → CH_3_SH + H_2_O (2)

2COS → CO_2_ + CS_2_ (3)

CS_2_ + 3H_2_ → CH_3_SH + H_2_S (4)

CH_3_SH + H_2_ → CH_4_ + H_2_S (5)

From Figure 1C, it can be seen that there was no significant difference in the main products formed over K-Mo/Al_2_O_3_ after being carbonized at different temperatures for the preparation of CH_3_SH from CO/H_2_/H_2_S; CH_3_SH, COS, CO_2_, CH_4_ and CS_2_ were the main products in this reaction system.

The H_2_-TPR curves of K-Mo/Al_2_O_3_ catalysts treated at different carbonization temperatures are shown in Figure 1D. The three materials with different carbonization temperatures exhibited similar reduction peak characteristics, with a low temperature reduction peak at 200–350 °C and a moderate temperature reduction peak at 450–600 °C. Moreover, the catalyst carbonized at 500 °C exhibited a reduction peak at a high temperature (above 700 °C), which was attributed to the reduction of Mo^4+^ to Mo^0^, and the hydrogen consumption peaks were obviously lower than those of the other two materials at the same reduction temperature. These results indicated that K-Mo/Al_2_O_3_ carbonized at 500 °C was more easily reduced under the same reduction conditions, resulting in more Mo with coordinatively unsaturated surface sites that were conducive to the adsorption of reactants and the desorption of intermediate products such as COS and CH_3_SH. Thus, the catalyst obtained by carbonization at 500 °C delivered the best catalytic performance, and the catalysts were carbonized at 500 °C in subsequent experiments.

### 3.2. The Role of Passivation

**Figure 2:**
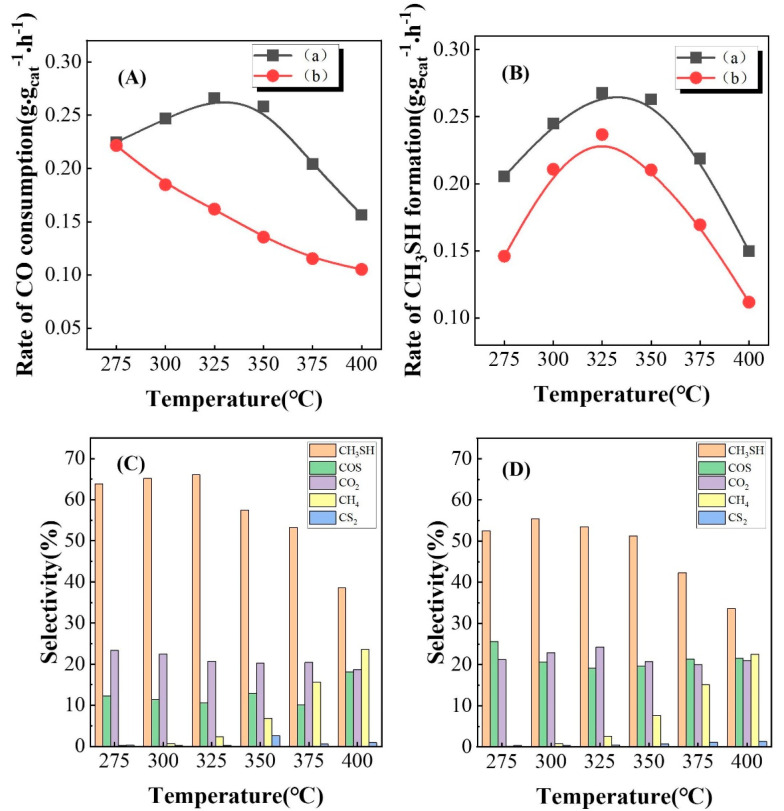
Catalytic activity of K-Mo_2_C/Al_2_O_3_ catalysts before and after passivation, (a) before passivation, (b) after passivation. Rate of CO consumption (**A**); Rate of CH_3_SH formation (**B**); Distribution diagram of unpassivated catalyst products (**C**); Distribution diagram of unpassivated catalyst products (**D**).

According to the literature, fresh molybdenum carbide materials are prone to violent reactions with air to form molybdenum oxide [38,39,40]. Therefore, fresh molybdenum carbide materials need to be passivated. After passivation, a passivation layer is formed on the surface of the material, which makes it more stable (passivation treatment with O_2_/inert gas). To reveal the role of passivation in the catalytic performance of K-Mo/Al_2_O_3_ for the synthesis of CH_3_SH from CO/H_2_/H_2_S, the catalytic activity of the catalysts before/after passivation was conducted. As shown in Figure 2, there was a significant change in the CO consumption rate within the reaction temperature range of 275–400 °C after passivation that decreased with increasing temperature. However, the formation rate trend of CH_3_SH remained almost unchanged, first increasing and then decreasing with increasing temperature. After comparing with the product analysis diagram, it was found that the formation of main products before/after passivation did not change (CH_3_SH, COS, CO_2_, CH_4_, and CS_2_), but the selectivity towards CH_3_SH was significantly reduced and the selectivity of by-product COS was increased after passivation. These changes were likely due to the passivation layer on the passivated catalyst surface, which covered the active site for direct hydrogenation of some COS to CH_3_SH, resulting in the reduction of selectivity towards CH_3_SH. The result revealed that passivation treatment was not effective for the synthesis of CH_3_SH from CO/H_2_/H_2_S, as the existing of the passivation layer would inhibit the formation of CH_3_SH.

### 3.3. Performance of Catalysts Carbonized under Different Atmospheres

**Figure 3:**
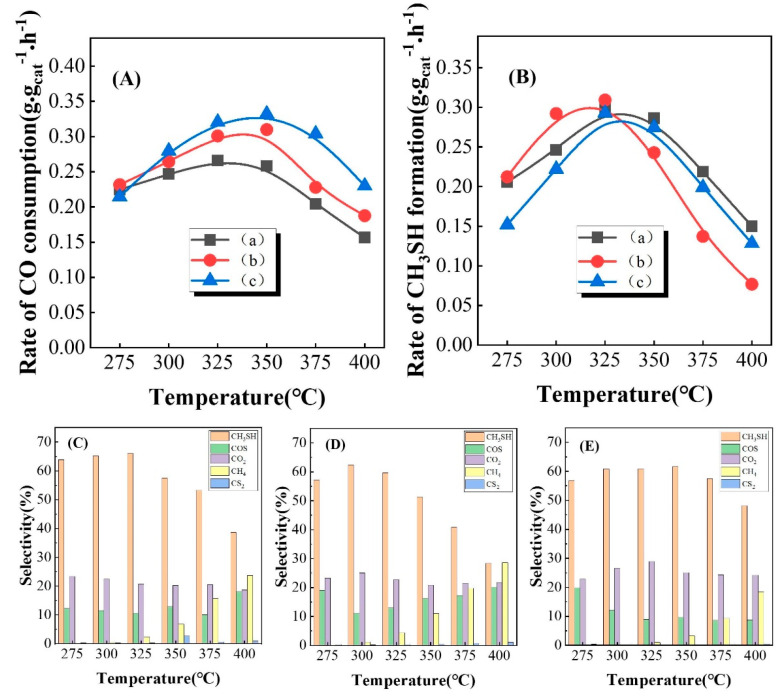
Effect of different carbonization atmospheres on the catalytic performance of K-Mo_2_C/Al_2_O_3_ catalysts, (a) CH_4_/H_2_, (b) C_2_H_6_/H_2_, (c) C_3_H_8_/H_2_. Rate of CO consumption (**A**); Rate of CH_3_SH formation (**B**); Distribution diagram of carbonization catalyst products in CH_4_/H_2_ atmosphere (**C**); Distribution diagram of carbonization catalyst products in C_2_H_6_/H_2_ atmosphere (**D**); Distribution diagram of carbonization catalyst products in C_3_H_8_/H_2_ atmosphere (**E**).

Different carbonization atmospheres can lead to the formation of different crystal forms of molybdenum carbide. For example, MoO_3_ will be converted into cubic molybdenum carbide with H_2_/toluene as the reaction gas at 673 K [41]; using H_2_/butane as the reaction gas, MoO_3_ is initially converted into face-centered cubic (fcc) molybdenum carbide, then the face-centered cubic molybdenum carbide gradually transforms into a hexagonal closely packed structure (fcp) with an increase in carbonization temperature [42]. Using alkanes with different carbon atomic numbers as a carbonization gas will generally lead to different carbonation degrees and then generate different numbers of active sites [27]. K-Mo/Al_2_O_3_ was carbonized by different atmospheres (CH_4_/H_2_, C_2_H_6_/H_2_, C_3_H_8_/H_2_), and the catalytic performance of different catalysts in the synthesis of CH_3_SH from CO/H_2_/H_2_S is shown in Figure 3. Remarkably, K-Mo/Al_2_O_3_ samples treated with different atmospheres all showed similar tendencies of catalytic performance, whereby the CO consumption rate first increased and then decreased. Catalysts treated with C_3_H_8_/H_2_ had the highest CO consumption rate, followed by catalysts treated with C_2_H_6_/H_2_, and catalysts treated with CH_4_/H_2_ had the worst CO consumption rate. From Figure 3B, it can be seen that the CH_3_SH generation rate of the catalyst treated with C_3_H_8_/H_2_, which had the highest CO consumption rate, was not optimum. Combined with the product analysis (Figure 3E), the selectivity towards by-product CO_2_ of this catalyst was high, which might have been caused by the hydrolysis of COS or the water–gas shift reaction of CO. Compared with that of the K-Mo_2_C/Al_2_O_3_ catalyst treated with C_3_H_8_/H_2_, the CO consumption rate of the K-Mo_2_C/Al_2_O_3_ catalyst treated with C_2_H_6_/H_2_ was slightly lower. It can be seen intuitively in Figure 3B that the CH_3_SH generation rate decreased sharply at high temperatures. And it was found that the selectivity of by-products of the catalyst was higher at high temperatures (COS, CH_4_) and the CH_3_SH selectivity was lower, which might be due to easy carbon deposition on the catalyst surface at high temperatures, further leading to a decrease in COS hydrogenation to generate CH_3_SH and excessive hydrogenation of CH_3_SH to generate CH_4_; thus, CH_3_SH selectivity decreased. Although the K-Mo_2_C/Al_2_O_3_ catalyst treated with CH_4_/H_2_ exhibited a poor CO consumption rate, it had a relatively stable CH_3_SH generation rate and high CH_3_SH selectivity. To reveal the role of carbonization atmospheres in catalytic performance, the characterization of catalysts was conducted.

### 3.4. Characterization of Catalysts Carbonized under Different Atmospheres

**Figure 4:**
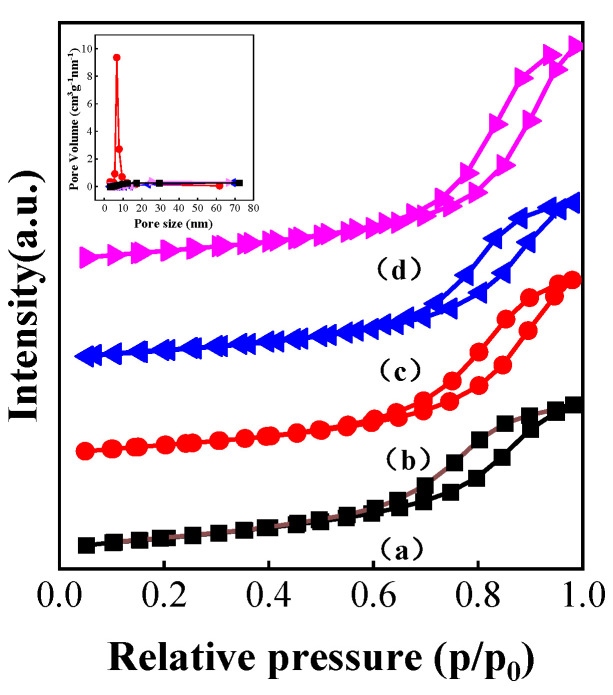
N_2_ adsorption–desorption isotherms of oxidation K-Mo/Al_2_O_3_ and Mo_2_C/Al_2_O_3_ samples treated in different carbonization atmospheres, (a) oxidized, (b) CH_4_/H_2_, (c) C_2_H_6_/H_2_, (d) C_3_H_8_/H_2_.

The N_2_ adsorption–desorption isotherms and physical properties of the catalysts assessed using N_2_ physisorption are displayed in Figure 4 and Table 1. As shown in Figure 4, the oxidation state sample was a typical mesoporous structure with an IV curve and an H1 hysteresis ring, and the surface area and pore volume were 116.1 m^2^/g and 0.269 cc/g, respectively. After carbonization, the specific surface area of all samples decreased slightly, with CH_4_-K-Mo_2_C/Al_2_O_3_ decreasing to 103.9 m^2^/g, C_2_H_6_-K-Mo_2_C/Al_2_O_3_ decreasing to 101.3 m^2^/g and C_3_H_8_-K-Mo_2_C/Al_2_O_3_ decreasing to 113.4 m^2^/g. However, the average pore volume of CH_4_-K-Mo_2_C/Al_2_O_3_, C_2_H_6_-K-Mo_2_C/Al_2_O_3_ and C_3_H_8_-K-Mo_2_C/Al_2_O_3_ increased to 0.321 cc/g, 0.290 cc/g and 0.390 cc/g, respectively. It can be therefore be concluded that the small decrease in specific surface area and the increase in pore volume after carbonization were caused by the collapse of some small pores during the carbonization process.

**Figure 5:**
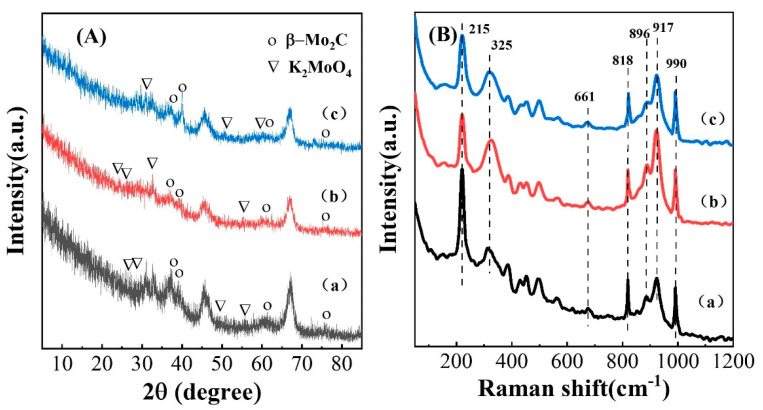
XRD (**A**) and Raman (**B**) patterns of K-Mo_2_C/Al_2_O_3_ samples under different carbonation atmospheres, (a) CH_4_/H_2_, (b) C_2_H_6_/H_2_, (c) C_3_H_8_/H_2_.

The phase compositions of the catalysts of different carbonization atmospheres were characterized by XRD and Raman spectroscopy. As shown in Figure 5A, the diffraction peaks at 2θ = 38.1°, 39.4°, 61.7° and 75.7° were attributed to β-Mo_2_C (JCPDS card No.65-8766) and correspond to the (002), (101), (110) and (201) crystal planes, respectively. Small diffraction peaks of K_2_MoO_4_ species (JCPDS card No.29-1021) were also detected. In the Raman spectrum (Figure 5B), diffraction peaks were detected at 325 cm^−1^, 896 cm^−1^ and 917 cm^−1^, which were attributed to monomolybdate MoO_4_^2−^ species and K_2_MoO_4_ on the sample surface [43,44], indicating an interaction between the precursor potassium and molybdenum. The diffraction peak was detected at 215 cm^−1^, assigned to well-dispersed AlMo_6_O_24_H_6_^3+^ anderson heteropolymetalate anions (AlMo_6_) formed by an alumina carrier and molybdenum-based aqueous solution [45,46,47]. The diffraction peaks at 661 cm^−1^, 818 cm^−1^ and 990 cm^−1^ were also assigned to β-Mo_2_C [48,49]; among these, the peaks at 818 cm^−1^ and 990 cm^−1^ correspond to the stretching vibrations of Mo-C-Mo and Mo-C, respectively. The results of XRD and Raman spectroscopy showed that there was no significant difference in the phase structure of the catalysts under different carbonization atmospheres.

**Figure 6:**
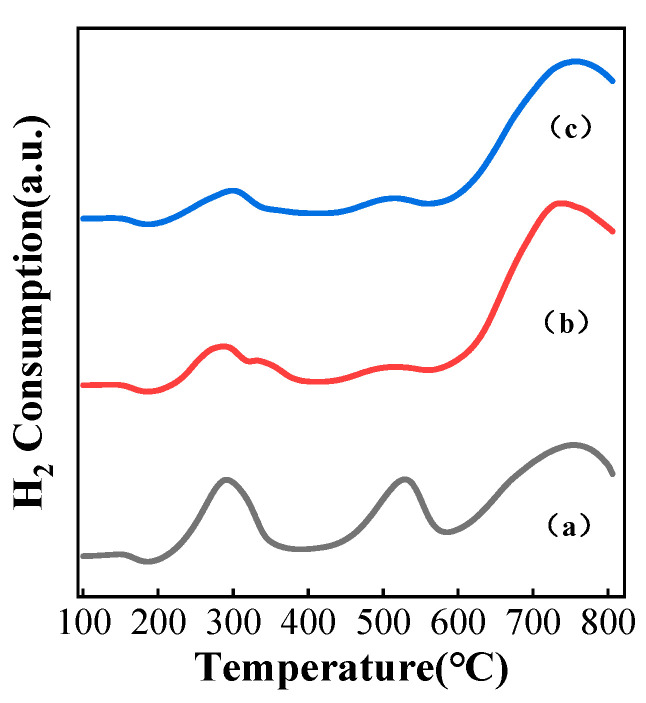
The H_2_-TPR spectra of K-Mo_2_C/Al_2_O_3_ samples in different carbonation atmospheres, (a) CH_4_/H_2_, (b) C_2_H_6_/H_2_, (c) C_3_H_8_/H_2_.

H_2_-TPR was carried out on the catalysts under different carbonization atmospheres to investigate redox properties; the results for the catalysts are displayed in Figure 6. It can be seen that all K-Mo_2_C/Al_2_O_3_ catalysts had three obvious hydrogen consumption peaks, two of which appeared at 200–400 °C and 400–600 °C, with larger hydrogen consumption peaks at >600 °C. Generally, the hydrogen consumption peak at low temperatures (200–400 °C) was the reduction of the passivation layer on the surface of the molybdenum carbide [50]. The moderate temperature reduction peak (400–600 °C) can be attributed to the reduction of Mo^6+^ to Mo^4+^ or other high-valent Mo species [51]. The high-temperature reduction peak (>600 °C) can be attributed to the reduction of Mo^4+^ or Mo^2+^ to Mo atoms [52]. However, the sample was not subjected to passivation treatment, and the presence of Mo^6+^ was not detected in the XRD spectrum. According to the literature [39,52], the hydrogen consumption peak at low temperatures (200–400 °C) is the reduction of surface high-valent molybdenum oxides, while the consumption peak at 650–800 °C is the reduction of Mo^4+^ or Mo^2+^ to Mo atoms. Therefore, in this work, the three hydrogen consumption peaks of 200–400 °C, 400–600 °C and >600 °C can attributed to the reduction of Mo^4+^, the reduction of potassium carbide species and the reduction of Mo^2+^ to Mo atoms, respectively. K-Mo_2_C/Al_2_O_3_ catalysts synthesized under different carbonization atmospheres exhibited similar H_2_-TPR reduction peaks, but the CH_4_-K-Mo_2_C/Al_2_O_3_ catalyst showed larger hydrogen consumption peaks at low and medium temperatures, indicating that more Mo coordinatively unsaturated surface sites were formed on the catalyst, which is more conducive to the adsorption of reactants and the desorption of products, consistent with the above catalytic performances.

**Figure 7:**
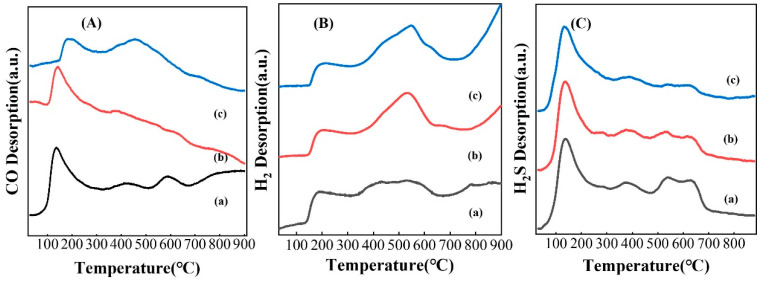
CO-TPD (**A**), H_2_-TPD (**B**) and H_2_S-TPD (**C**) spectra of (a) CH_4_-K-Mo_2_C/Al_2_O_3_, (b) C_2_H_6_-K-Mo_2_C/Al_2_O_3_, (c) C_3_H_8_-K-Mo_2_C/Al_2_O_3_ samples.

The TPD technique is commonly used to characterize the surface properties and chemical reactions of a material. As an important step in a catalytic reaction, the adsorption of reactant molecules by the catalyst plays a crucial role in the catalytic performance [53,54]. To investigate the adsorption and activation abilities of K-Mo_2_C/Al_2_O_3_ catalysts under different carbonization atmospheres for reactants, the catalysts were characterized by carbon monoxide, hydrogen and hydrogen sulfide adsorption and desorption; the results of CO-TPD, H_2_-TPD and H_2_S-TPD of the catalysts are displayed in Figure 7. As shown in Figure 7A, the CH_4_-K-Mo_2_C/Al_2_O_3_ catalyst exhibited three CO desorption peaks, indicating three different CO adsorption sites and on the catalyst with different adsorption strengths. According to the literature, CH_4_-K-Mo_2_C/Al_2_O_3_ has a low-temperature desorption peak at around 120 °C, which was assigned to the desorption of CO and physical adsorption of Mo_2_C. In the high-temperature region, the desorption peak was the dissociation adsorption peak formed by the strong chemical adsorption between the catalyst and CO [52]. C_2_H_6_-K-Mo_2_C/Al_2_O_3_ and C_3_H_8_-K-Mo_2_C/Al_2_O_3_ showed one and two CO desorption peaks, respectively, which were obviously lower than those of CH_4_-K-Mo_2_C/Al_2_O_3_. According to the related research, the Mo coordinatively unsaturated sites serve as the adsorption sites for the activation of CO [6,7,55]. Therefore, the CH_4_/H_2_ atmosphere promotes the generation of more Mo coordinatively unsaturated sites on the catalyst and thus facilitates CO adsorption and activation, which also enhances the selectivity of CH_3_SH.

In H_2_-TPD, the CH_4_-K-Mo_2_C/Al_2_O_3_ catalyst exhibited slightly strong hydrogen activation ability, producing a large amount of desorbed H at medium temperature, and there was an obvious H_2_ negative peak, indicating that hydrogen might be dissociated into adsorbed H* at this temperature. Moreover, as shown in the H_2_S-TPD results (Figure 7C), the CH_4_-K-Mo_2_C/Al_2_O_3_ catalyst showed that it had strong H_2_S adsorption and dissociation abilities, very similar to the other two catalysts in the low temperature range but slightly stronger above 500 °C. In short, the use of CH_4_/H_2_ as a carbonization atmosphere facilitates the adsorption and activation of reactants of K-Mo_2_C/Al_2_O_3_, thus delivering a high activity and selectivity towards CH_3_SH.

## 4. Conclusions

In conclusion, a series of K-Mo_2_C/Al_2_O_3_ catalysts were successfully obtained through the impregnation method with a carbonization process at temperatures of 400–600 °C. Through careful control of the carbonization temperature, passivation process and carbonization atmosphere (500 °C, unpassivated and CH_4_/H_2_ being optimal), a CH_4_-K-Mo_2_C/Al_2_O_3_ catalyst with unusually high activity and selectivity towards CH_3_SH produced from CO/H_2_/H_2_S at 325 °C could be obtained (e.g., 66.1% selectivity and 0.2990 g·g_cat_^−1^·h^−1^ formation rate to CH_3_SH). Structural characterization studies revealed that passivation of the catalysts would lead to a layer formed on the surface of the material, covering the active sites of the reaction and reducing the activity of the catalyst. Under different carbonization temperatures and atmospheres (CH_4_/H_2_, C_2_H_6_/H_2_, C_3_H_8_/H_2_), the catalyst carbonized by CH_4_/H_2_ at 500 °C exhibited higher catalytic activity because it was more easily reduced under the same reduction conditions and resulted in more Mo coordinatively unsaturated sites, which was conducive to the adsorption of reactants and the desorption of intermediate products, thus showing the best overall performance. This work identifies K-Mo_2_C/Al_2_O_3_ as a promising new Mo-based catalyst for the production of CH_3_SH from CO/H_2_/H_2_S.

## Figures and Tables

**Table 1:** Textural characteristics of oxidation K-Mo/Al_2_O_3_ and Mo_2_C/Al_2_O_3_ samples treated in different carbonization atmospheres.

Sample	Surface Area (m^2^/g)	Pore Volume (cc/g)	Pore Diameter Dv (d) (nm)
K-Mo/Al_2_O_3_	116.1	0.269	7.853
CH_4_-K-Mo_2_C/Al_2_O_3_	103.9	0.321	9.618
C_2_H_6_-K-Mo_2_C/Al_2_O_3_	101.3	0.290	8.598
C_3_H_8_-K-Mo_2_C/Al_2_O_3_	113.4	0.390	11.159

## Data Availability

All data used to support the findings of this study are included within the article.
